# Single-cell nuclear RNA-sequencing reveals dynamic changes in breast muscle cells during the embryonic development of Ding’an goose

**DOI:** 10.1371/journal.pone.0338390

**Published:** 2025-12-11

**Authors:** Tieshan Xu, Yingxiu Hu, Haokai Fan, Xinli Zheng, Qicheng Jiang, Lizhi Lu, Jie Li, Zhemin Lin, Lihong Gu

**Affiliations:** 1 Institute of Animal Science & Veterinary Medicine, Hainan Academy of Agricultural Sciences, Haikou, China; 2 Tropical Crop Genetic Resource Research Institute, Chinese Academy of Tropical Agricultural Sciences, Haikou, China; 3 Hainan Institute, Zhejiang University, Yongyou Industry Park, Yazhou Bay Sci-Tech City, Sanya, China; 4 Zhejiang Academy of Agricultural Sciences, Hangzhou, Zhejiang, China; 5 Hainan Wansheng Animal Husbandry Development Co., Ltd, Wanning, China; Tarbiat Modares University, IRAN, ISLAMIC REPUBLIC OF

## Abstract

Breast muscle is a crucial trait in poultry meat production. Previous studies have identified embryonic day 15 (E15), E21, and E31 as key time points in the breast muscle development of Ding’an goose, yet the specific molecular mechanisms remain unclear. In this study, we analyzed cellular heterogeneity and molecular dynamics at 15th day of embryonic breast muscle of Dingan goose (E15), E21, and E31 by using single-nucleus RNA-sequencing (snRNA-seq) technology. Nine types of cells were discovered, including fibroblast adipogenic progenitor cells (FAPs), myocytes and muscle stem cells (MuSCs), with notable differences among the three developmental stages in terms of cell type and abundance: FAPs and MuSCs gradually decreased from 42.3% and 35.6% (E15) to 15.7% and 12.2% (E31), respectively, while myocytes increased from 18.5% (E15) to 70.1% (E31). Additionally, the distinct heterogeneity of myocytes, MuSCs and FAPs was determined based on the analysis of gene regulatory networks for each cluster. Developmental trajectory analysis identified genes related to the function and development of MuSCs. The identified differentially expressed genes elucidate the molecular mechanisms of cellular dynamic changes during breast muscle development. This study generated a nuclear profile for single muscle cells that played a key role in the development of breast muscle in Ding’an goose embryos. We investigated metabolic changes at the cellular level during three key developmental stages, thereby refining our understanding of the molecular mechanisms underlying pectoral muscle development specifically in embryonic Ding’an goose.

## 1. Introduction

Skeletal muscle accounts for approximately 35–60% of body weight in animals [1], which represents a vital trait for the production of poultry meat. Breast muscles are involved in functions such as muscle movement, energy expenditure, and endocrine regulation [2]. Thus, it is a crucial tissue for human and animals. The formation of skeletal muscle involves multiple biological processes such as the proliferation, migration and differentiation of skeletal muscle stem cells, the proliferation and differentiation of myoblasts [3,4]. Almost all muscle fibers have already differentiated and formed during the embryonic stage, and only hypertrophy of muscle fibers and transformation of muscle fiber types occur after birth [5]. Understanding the developmental mechanisms of embryonic breast muscle is therefore crucial for enhancing goose meat yield.

RNA-sequencing (RNA-seq) is widely used to identify regulatory genes in various tissues or organs [6–9], and has significantly promoted the exploration of mechanisms underlying animal economic traits over the past decade. In avian embryonic muscle research, RNA-seq has been applied to screen key regulators in chickens and ducks. For example, studies on broiler chickens identified MYOD1, MYOG and PAX7 as core genes controlling embryonic breast muscle differentiation [10]; in Pekin ducks, RNA-seq analyses linked the Wnt/β-catenin signaling pathway to the proliferation and differentiation of embryonic muscle cells [11]. However, bulk RNA-seq averages transcriptomic signals across cell populations, failing to clearly distinguish changes specific to cell types or subtypes [12], these limitation hinder the study of cell-specific regulatory mechanisms in breast muscle development.

Single-nucleus RNA-sequencing (snRNA-seq) overcomes this drawback by characterizing transcriptomes in the nuclei of individual cells at different developmental stages [13]. This technology enables the analysis of gene expression heterogeneity between cells, the tracking of cell lineage trajectories during development, and the identification of cell-type-specific gene expression [14], making it increasingly widely used in embryonic development research of species such as mice and chickens [15,16]. For instance, snRNA-seq in chicken embryos revealed the heterogeneity of MuSCs and identified subtype-specific regulatory genes during myogenesis [17].

In goose research, however, studies on embryonic breast muscle development remain relatively scarce. Existing studies primarily focus on post-hatch growth. For example, a study on Landes geese found that the mTOR signaling pathway regulates breast muscle hypertrophy by promoting protein synthesis in post-hatch stages [18]; another study on Zhedong white geese explored the relationship between genes related to lipid metabolism and intramuscular fat deposition in post-hatch breast muscle [19]. For embryonic stages, only a few bulk RNA-seq studies have been reported. Our previous study [20] has shown that myogenic differentiation is the major event for the earlier stages and peaks at the 15th day (E15) during the embryonic development of Ding’an goose. After that, the myogenic differentiation slows down, muscle fiber fusion appears and and peaks at the 21st day (E21) during the embryonic development of Ding’an goose, With the muscle fiber development of Dingan goose, it arrives its mature muscle fiber point at the 31st day (E31) during the embryonic development. Thus, E15, E21 and E31 represent three key time points for breast muscle development, which will provide many key events in the regulation of breast muscle development for Ding’an goose. To date, no studies have applied snRNA-seq to analyze goose embryonic breast muscle, resulting in a lack of understanding of cellular heterogeneity and cell-type-specific regulatory mechanisms during its development.

Ding’an goose is the only local meat goose variety in Hainan Province, and was listed in the National Livestock and Poultry Genetic Resources List in 2010. It has the characteristics of good adaptability, strong stress resistance, high lean meat percentage, and good meat quality, making it an important component of animal husbandry in Hainan Province [20]. To fill the aforementioned research gap, we used snRNA-seq technology to analyze breast muscle cells of Ding’an geese at E15, E21, and E31. The aim of this study is to clarify the dynamic changes and heterogeneity of breast muscle cell populations, and to identify cell-type-specific regulatory genes and pathways, thereby enhancing the understanding of the molecular mechanisms underlying embryonic breast muscle development in Ding’an goose.

## 2. Methods

### 2.1. Sample collection

The fertilized eggs of Ding’an goose are provided by Minqiong Poultry Industry Co., Ltd (Hainan, China). All animal experiments were approved by the Animal Ethics Committee of IASVM-HAAS (Approval No.:IASVMHAAS-AE-202489). Fertilized eggs of Ding’an goose were incubated in an incubator at 38°C and 70% relative humidity. At embryonic days 15 (E15), 21 (E21), and 31 (E31), embryos were sacrificed humanely following the AVMA Guidelines for the Euthanasia of Animals (2020).

Embryos were anesthetized via inhalation of 5% isoflurane in oxygen (flow rate: 1 L/min) until the absence of toe-pinch reflex was confirmed. Euthanasia was then performed by cervical dislocation to ensure rapid and painless death. To minimize suffering, all operations were conducted by trained personnel, and embryos were handled gently to avoid unnecessary stress. Incubation and sacrifice procedures were designed to reduce exposure time to non-physiological conditions, and sterile techniques were used during tissue collection to prevent post-mortem contamination.

At the time points of E15, E21, and E31, the breast muscles were aseptically harvested. The left breast muscles were sent to LC Bio Technology Co. Ltd. (Hangzhou, China) for snRNA-seq. Half of the right breast muscles were fixed in 4% paraformaldehyde for immunofluorescence experiments, while the other half were used for fluorescence quantitative PCR experiments. Three individual replicates were set up for each time point.

### 2.2. Single-nuclei isolation

Nuclei were isolated with Nuclei EZ Lysis buffer (NUC-101; Sigma-Aldrich) supplemented with protease inhibitor(5892791001; Roche) and RNase inhibitor(N2615; Promega and AM2696; Life Technologies). Samples were cut into 1 mm pieces and homogenized using a Dounce homogenizer (885302-0002; Kimble Chase) in 2 ml of ice-cold Nuclei EZ Lysis buffer. They were incubated on ice for 5 minutes with an additional 2 ml of lysis buffer. Grounded with a dounce(Sigma), resuspended by pipette, gentlely. Incubated on ice for 6 min, then add 2 ml of ice-cold 4% BSA, resuspended by Pasteur pipette, then stop the reaction. Centrifuged at 300g for 5 minutes at 4°C. Add 2 mlof lysis buffer and 4% BSA, resuspended, Incubated on ice for 3 min. De-fragment with Miltenyi (Debris Removal Solution). The pellet was resuspended and washed with 4 ml of the buffer, and then, it was incubated on ice for 5 minutes. After another centrifugation. The pellet was resuspended in Nuclei Suspension Buffer (1x PBS,0.07% BSA, and 0.1% RNase inhibitor). Filtered through a 20-mm cell strainer(43-50020-50; pluriSelect), and counted using a haemocytometer/ Countess II Automated Cell Counter and concentration adjusted to 700–1200 cells/μl.

### 2.3. Library preparation and sequencing

Single-nuclei suspensions were loaded to 10x Chromium to capture 8000 single cell according to the manufacturer’s instructions of 10X Genomics Chromium Single-Cell 3’ kit (V3). The following cDNA amplification and library construction steps were performed according to the standard protocol [21]. Libraries were sequenced on an Illumina NovaSeq 6000 sequencing system (paired-end multiplexing run,150 bp) by LC-Bio Technology co.ltd., (HangZhou,China) at a minimum depth of 20,000 reads per cell.

### 2.4 Bioinformatics analysis

First, we used Illumina bcl2fastq software (version 5.01) to multiplex the sequencing results and convert them to FASTQ format. The Cell Ranger pipeline was used for sample demultiplexing, barcode processing, and single-cell 3’-gene counting. Next, snRNA-seq data were aligned with the Ensembl genome goose reference genome, and the Cell Ranger output was loaded into Seurat (version 3.1.1) for dimensionality reduction, clustering, and the analysis of snRNA-seq data [22]. Overall, 25000 cells passed the quality control threshold in that any genes expressed in < 3 cells (default parameter: 1 cell) were removed, the number of genes expressed in each cell > 500 was low, < 5000 was high, the UMI count was < 500, and the percentage of mitochondrial DNA derived gene expression was < 25% [23]. To visualize the data, we used Seurat software to further reduce the dimensionality of all 18339 cells, and t-SNE was used to project the cells into a two-dimensional (2D) space [24]. Then, we used the LogNormalize method in the normalization function of Seurat to calculate gene expression values, and perform principal component analysis on normalized gene expression values. Then, we used cluster and t-SNE analysis to analyze the top 10 principal components [25]. To identify clusters, we selected a clustering method based on a weighted shared nearest neighbor graph. Marker genes for each cluster were identified using the the FindAllMarkers function in Seurat and Wilcoxon’s rank sum test (default parameter: “bimodal”:likelihood ratio test) with default parameters. This process selected marker genes that were expressed in > 10% of cells in the cluster and had a mean log (fold change) > 0.25 (default parameter: 0.26) [26].

### 2.5. Immunofluorescence staining

The tissues used for immunofluorescence was fixed with an environmentally friendly memory muscle fixative (Servicebio, Hubei, China) for 24 hours. The tissues were dehydrated, embedded, and sectioned for analysis, as described previously [27]. Then, we performed immunohistochemistry with a specific polyclonal antibody (1:200) to detect the spatial distribution of PAX7 (GB113190−50, Servicebio, Hubei, China) protein in skeletal muscle cells, which has been validated in previous studies [28]. Next, we incubated sections with a HRP-labeled secondary antibody (Servicebio, Hubei, China) (1:1000) and counterstained with DAPI (Beyotime, Shanghai, China) (1:1000).

### 2.6. Fibroblast extraction

For tissues used for fluorescence quantitative PCR, cut them into small pieces with scissors, and digested them with 4% trypsin at 37 °C for one hour. The final cell suspension was then filtered through a 200µm cell filter, centrifuged at 1800 rpm for 10 minutes, washed with PBS, and re-centrifuged for 5 minutes. Adherent cells were collected one hour later.

### 2.7. Fluorescence quantitative polymerase chain reaction (PCR)

We extracted total RNA from fibroblasts using the Trizol method (Invitrogen, Carlsbad, CA, USA), and measured the concentration of the total RNA with a UV spectrophotometer (A260/280: 1.8–2.0). Then, we used a Tiangen Reverse Transcription Kit (Beijing, China) to synthesize c-DNA for PCR [19]. Primers (Table 1) were designed and synthesized by Shenggong Bioengineering Co., Ltd. (Shanghai, China). GAPDH is widely validated and used as a reference gene in avian muscle development studies [29]. For PCR, we used c-DNA as a template and GAPDH as an internal reference, and fluorescence quantitative PCR system. This allowed us to determine the gene expression by the 2^-ΔΔCt^ method [30]. Three biological replicates and three technical replicates were performed. Experimental data are expressed as mean ± standard error of the mean. Data were compared with the Student’s t-test, and a P < 0.05 was considered significant.

**Table 1:** Primer sequences for real time qPCR.

NCBI number	Gene name	Sequence 5’-3’	Product length (bp)
XM_048079166.2	FOXO3-F	TGCCTTGTCCAATTCCATCAGTAAC	117
	FOXO3-R	GAGAGCGGGTCAGAAAGTGTTTG	
XM_066995163.1	DCN-F	CTATATCCGTATCGCAGACACCAAC	82
	DCN-R	ACCATCAAGATGAAGTTCCGTAAGG	
XM_013172072.3	PLXDC2-F	CCACAGAAGATGACACCAAGATAGC	111
	PLXDC2-R	CAACAATTAAGCCAGCGTGAAGTG	
XM_013178763.3	COL3A1-F	TGGAGAGTCTATGAATGGAGGCTTC	109
	COL3A1-R	GGCACGGCTGGAGAGGATG	
XM_067000417.1	ABLIM1-F	AACCTCTTCTCTTCCTGGCTATGG	105
	ABLIM1-R	TCTCTAACACCACCACTCACATCC	
XM_048066782.2	EGFL6-F	TTACCGACTTGCTGGCGAGAG	106
	EGFL6-R	TCCACCTTTCATCTTTCCCTTTGTTC	
XM_067004670.1	GAPDH-F	GTAGTGAAGGCTGCTGCTGATG	106
	GAPDH-R	CAAAGGTGGAGGAATGGCTGTC	

## 3. Results

### 3.1. Identification of different cell types

**Fig 1:**
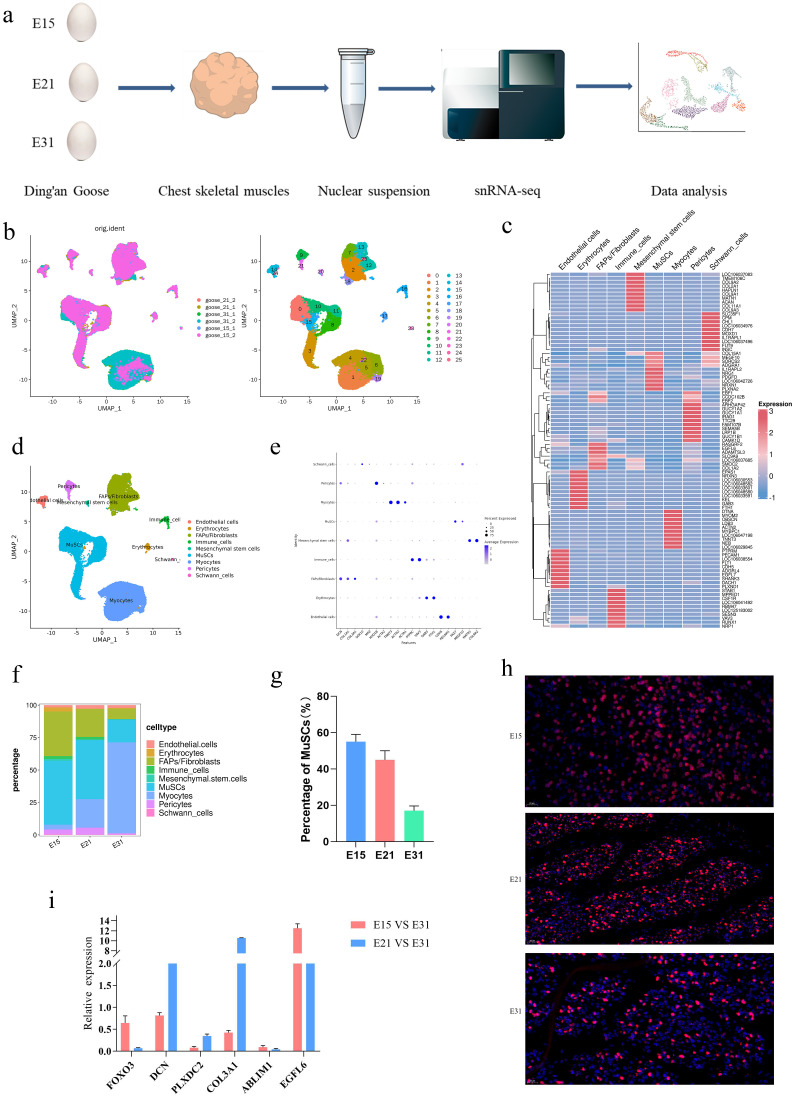
Analysis of different cell types in the breast muscle of goose. (a) Single-cell nuclear sequencing process. (b) 64852 single-cell reduced dimensional maps based on T-distribution random neighborhood embedding (t-SNE), with different colors representing different cell clusters (the same color system applies for all other Figs). (c) Heat map showing the top ten upregulated genes in each cell cluster. (d) t-SNE map of cells identified in the breast muscle. (e) t-SNE plot showing the expression of marker genes in fibroblast adipogenic progenitor cells, Schwann cells, pericytes, myocytes, immune cells, red blood cells, endothelial cells, MuSCs, and mesenchymal stem cells. (f) Proportional diagram showing cells at different developmental stages. (g) Immunofluorescence staining of MuSCs, blue staining (DIPI) represents all cell nuclei, and red staining (PAX7) represents myoblasts. (h) Staining analysis of MUSC immunofluorescence. (i) qPCR analysis of the expression levels of certain DEGs in fibroblasts. Error bars represent the standard error of the mean (SEM); n = 3 biological replicates, with 3 technical replicates per biological replicate, the same below.

In order to generate a cell population map for developing breast muscle in goose, we performed snRNA-seq on breast muscle tissues from goose embryos on E15, E21, and E31 (Fig 1a). After Seurat filtering, we collected 18972, 19460, 19843 single cells from E15, E21, and E31 respectively, and resulting in the generation of 26 clusters (Fig 1b). A heatmap showing the top 10 upregulated genes in each of these 26 clusters is presented in Fig 1c, which reveals cluster-specific gene expression patterns that facilitate subsequent cell type annotation. Based on the enriched the differentially expressed genes (**DEGs**) (Additional file S1 Table) and cell-type specific gene markers in each cluster, nine cell populations were identified (Fig 1d), including fibroblast adipogenic progenitor cells/fibroblasts (FAPs, clusters 2, 7, 12, 13, 18, and 25; Marker genes: PDGFRα, CD34), Schwann cells (SCs, cluster 23; Marker gene: MPZ), pericytes (Peri, clusters 9 and 21; Marker gene: PDGFRβ), muscle cells (Myocyte, clusters 1, 4, 5, 6, 19, and 22 Marker genes: MYOD1, MYH1), immune cells (ICs, cluster 16; Marker gene: CD45), red blood cells (RBCs, cluster 17; Marker gene: HBB), endothelial cells (ECs, clusters 14 and 24; Marker gene: CD31), muscle stem cells (MuSCs, clusters 0, 3, 8, 10, 11, and 15; Marker gene: PAX7), and mesenchymal stem cells (cluster 20; Marker gene: CD73). The distributions of cells expressing characteristic gene markers were shown in Fig 1e. In addition, we identified key differences in the abundance of cell clusters for E15, E21, and E31 (Fig 1f). FAPS/fibroblasts, MuSCs, and myocytes were the main constituent cells of breast muscle (over 85%) at three time points. As time progressed, the proportion of FAPS/fibroblasts and MuSCs were decreased (from 35% to 8% and 55% to 20%, respectively), while the proportion of myocytes is increased (from 5% to 70%). For E31, myocytes accounted for the largest cell proportion among the nine cell types (70%). To verify the sequencing results, we conducted immunofluorescence experiments and found that the proportion of MuSCs was consistent with the snRNA-seq results (Fig 1g and Fig 1h). We used qPCR to investigate the expression levels of certain DEGs in fibroblasts and found that the qPCR results were consistent with that of snRNA-seq data (Fig 1i), thus demonstrating the reliability of our sequencing results. Considering the proportion of composition and developmental trends in breast muscles, we further analyzed FAPS/fibroblasts, MuSCs, and myocytes.

### 3.2. Transcriptional heterogeneity of MuSCs

**Fig 2:**
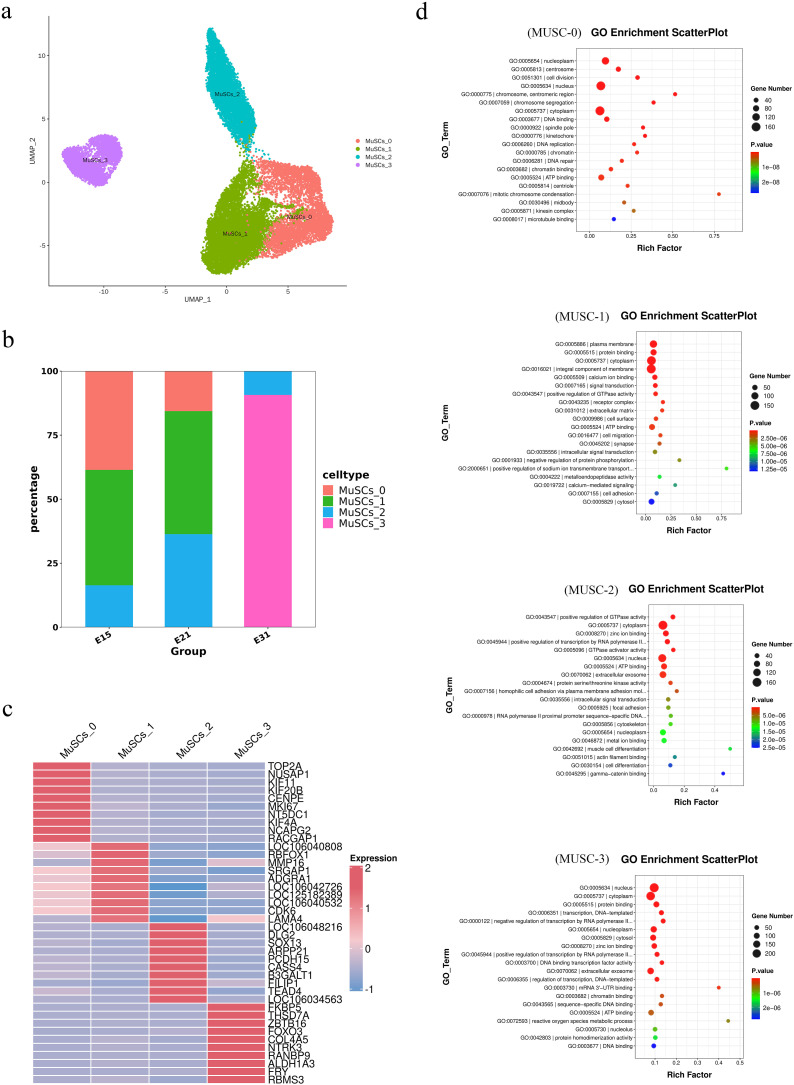
SnRNA-seq analysis showing the expression levels of different genes in MuSCs extracted from the embryonic breast muscle of goose. (a) t-SNE map showing MUSC subgroups. (b) The proportion of MUSC subgroups at E15, E21, and E31 developmental stages. (c) Heat map showing the top 10 upregulated genes in each subgroup of MuSCs. (d) GO functional diagram showing MuSCs subgroups.

To investigate the heterogeneity of MUSC, we divided MUSC into 12 clusters and further analysis revealed these cell clusters exhibited characteristics of four main cell sub-types (Fig 2a), including MUSC-0, MUSC-1, MUSC-2, and MUSC-3 (Fig 2b). Next, we identified four MUSC sub-groups by characteristic genes (Fig 2c). MUSC-0 expressed higher levels of *TOP2A*, *MKI67*, and *CENPE*, indicating that the cell population was probably in a proliferative state. Genes related to cell migration, such as *MMP16*, *LAMA4*, and *SRGAP*1, were highly expressed in the MUSC-1. However, the MUSC-2 expressed higher levels of *SOX13*, *PCDH15*, *TEAD4*, and *FILIP1*. The MuSCs-3 cell cluster specifically expressed *ZBTB16*, *FOXO3*, and *COL4A5*. To further reveal the functionality of each MUSC sub-group, we performed functional enrichment for each MUSC sub-group (Fig 2d). Gene ontology (GO) analysis revealed enrichment in cell division, chromosome separation, and DNA binding in the MUSC-0 cell cluster. The MUSC-1 cluster was enriched in protein binding, signal transduction, and cell migration. The MUSC-2 cluster was associated with the transcriptional regulation of polymerase II and zinc ion binding while the MUSC-3 cluster was associated with protein binding and DNA binding transcription factor activity. Collectively, these results indicated that the transcriptional profiles of different subgroups of MuSCs undergo key changes during the development of embryonic breast muscle in goose, suggesting that they may serve different functions.

### 3.3. The transition trajectory of MuSCs

**Fig 3:**
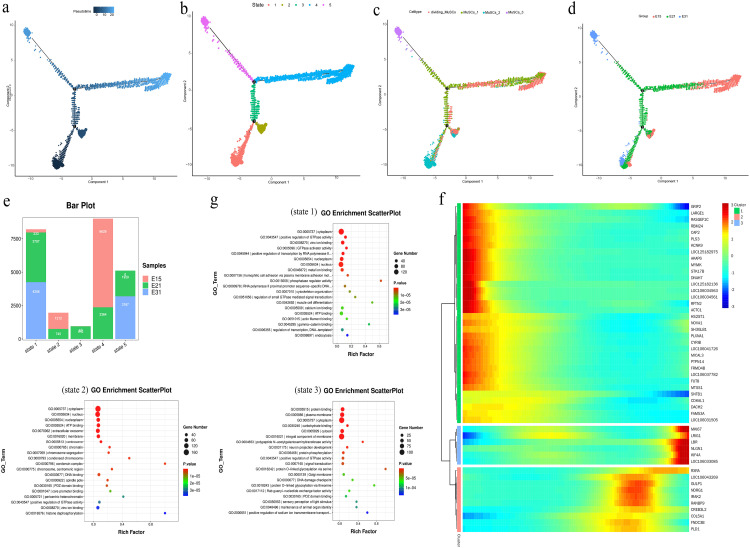
Pseudo-time analysis of the differentiation trajectory of MUSC subgroups. (a) Pseudo-time analysis of individual MuSCs subgroups. (b) Pseudo-time analysis of individual MuSCs subgroups. (c) Pseudo-time analysis based on MuSCs subgroups. (d) Pseudo-time analysis of MUSC subgroups based on three developmental stages. (e) The distribution of five states in three developmental stages. (f) Gene expression heatmap showing pseudo- temporal tree branches. (g) GO enrichment map corresponding to the fate of each cell.

In order to determine the developmental trajectory of MuSCs, we next performed pseudo-time analysis on all MUSC data using clusters based on the Monocle2 algorithm. The differentiation trajectory of MuSCs is shown in Fig 3a. As differentiation progressed, the MuSCs were classified into five states (Fig 3b), with distinct differentiation trajectories among subgroups and different developmental trajectories (Fig 3c and S1 Table). The cell sub-types in the early developmental stage were mainly composed of MUSC-0, while in the middle and late stages were predominantly MUSC-1, MUSC-2, and MUSC-3. In addition, we found that the cell sub-types at E15 were predominantly MUSC-0 and MUSC-1, MUSC-1 and MUSC-2 predominated at E21, and E31 was predominantly MUSC-3 (Fig 3d).

To further investigate the molecular mechanisms underlying the functional evolution of MuSCs, we used BEAM analysis to differentiate the identified branch specific DEGs and observed significant changes in the pseudo time function. According to pseudo-temporal expression patterns, genes were divided into five states (Fig 3e and 3f). Biological process analysis (Fig 3g) revealed that genes with high expression of States 1 and 2 were enriched in processes in the cytoplasm and nucleus, while State 3 was associated with cytoplasm and membrane components.

### 3.4. Typing of FAPs during embryonic development

**Fig 4:**
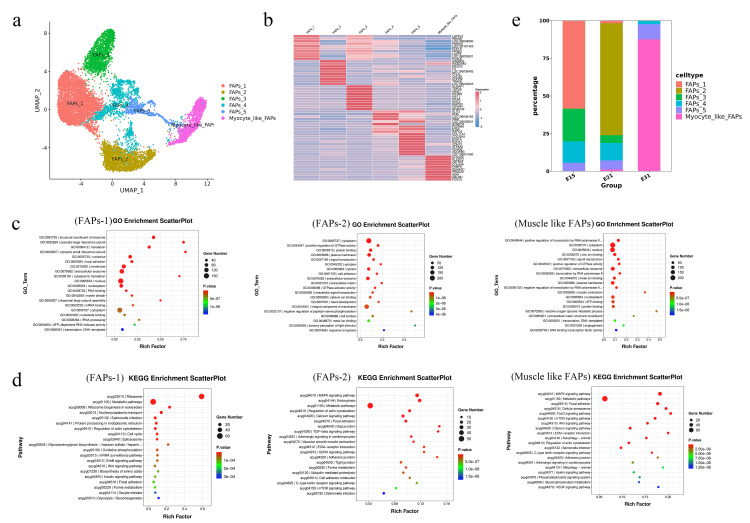
SnRNA-seq analysis showing the expression patterns of different genes in FAPs. (a) t-SNE map showing subgroups of FAPs. (b) Heatmap showing the top ten upregulated genes in the identified FAPs subgroups. (c) GO enrichment in different FAPs subgroups. (d) KEGG analysis of different FAPs subgroups. (e) The proportion of FAPs subgroups in three key developmental stages.

FAPs were divided into 13 clusters, and further analysis revealed that these cell clusters exhibited the characteristics of six main cell sub-types (Fig 4a), including FAPs-1, FAPs-2, FAPs-3, FAPs-4, FAPs-5, and muscle cell-like FAPs. FAPs-1 upregulated genes (Fig 4b) were mainly associated with the functionality of extracellular vesicles, cell membranes, and nuclei (Fig 4c), and also related to ribosomes and metabolic pathways (Fig 4d), in which they are known to participate in the cell cycle regulation of *EGFL6* overexpression [21]. FAPs-1 was closely related to cell proliferation and development. FAPs-2 upregulated genes were associated with several components, including the cytoplasm, membranes, and protein binding, and were enriched in endocytosis, metabolism, and MAPK signaling pathways. Of these, C-type lectin chondroitin lectin (*CHODL*) was clearly upregulated [22]. The main enrichment of muscle cell-like FAPs was in the nucleus and cytoplasm, and was associated with adhesion, metabolism, and MAPK signaling pathways. Of these, *FOXO3*, *PLXDC2*, and *ABLIM1* were specifically expressed, and muscle cell-like FAPs exhibited the characteristics of muscle cells. Next, we compared the proportion of cells at different developmental stages in each cluster (Fig 4e). FAPs-1 accounted for the highest proportion at stage E15, FAPs-2 accounted for the highest proportion at E21, and muscle cell-like FAPs accounted for the highest proportion at E31.

### 3.5. Typing of myocytes during embryonic development

**Fig 5:**
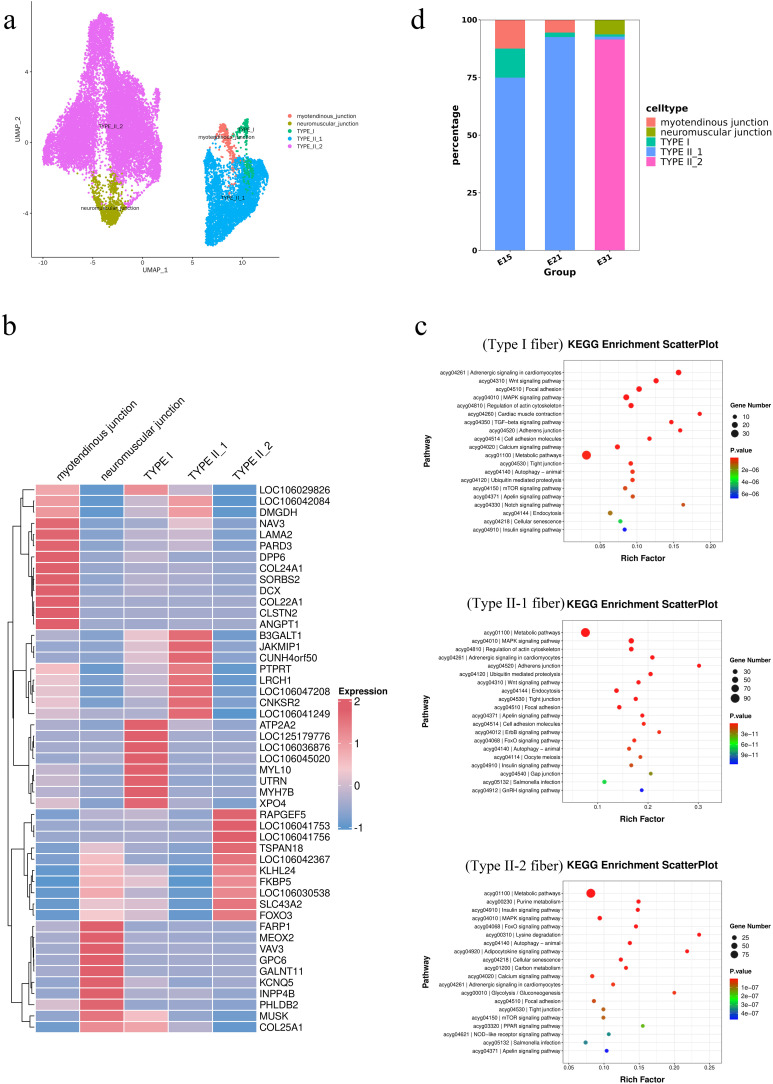
SnRNA-seq analysis showing the expression patterns of different genes in myocytes. (a) t-SNE map showing sub-populations of muscle cells. (b) Heatmap showing the top ten upregulated genes in the sub-populations of muscle cells. (c) KEGG analysis of differentially expressed genes in different subgroups of muscle cells. (d) Proportions of muscle cell sub-populations in three key developmental stages.

Myocytes were divided into 12 clusters, and further analysis revealed that these cell clusters exhibited the characteristics of five main cell types (Fig 5a), including neuromuscular junctions (NJs), tendon junctions (MJs), type I fibers, type IIa fibers, and type IIb fibers. As shown in Fig 5b, NJs expressed *ACHE* and *ETV5*; type I fibers expressed *TNNC1*, *ATP2A2*, *TNNI1*, *MYL10*, and *MYH7B*; type II-1 fibers expressed *LRCH1*, *CNKSR2*, *B3GALT1*, *JAKMIP1*, *TPM1*, and *TNNC2*; and type II-2 fibers expressed *FOXO3*, *FKBP5*, *RAPGEF5*, *TSPAN18*, and TNNT3. Next, we conducted functional enrichment analysis on type I fibers, type II-1 fibers, and type II-2 fibers (Fig 5c). The genes upregulated in type I fibers and II-1 fibers were mainly associated with metabolism, and the MAPK and Wnt signaling pathways. The genes enriched in type II-2 fibers were mainly associated with signaling pathways such as metabolism and purine metabolism. Of these three developmental stages (5d), type IIa fibers accoutned for the highest proportion at E15 and E21 (75% and 85%, respectively), while type IIb fibers accounted for the highest proportion at E31 (90%).

### 3.6. Breast muscle gene regulatory network dynamics

**Fig 6:**
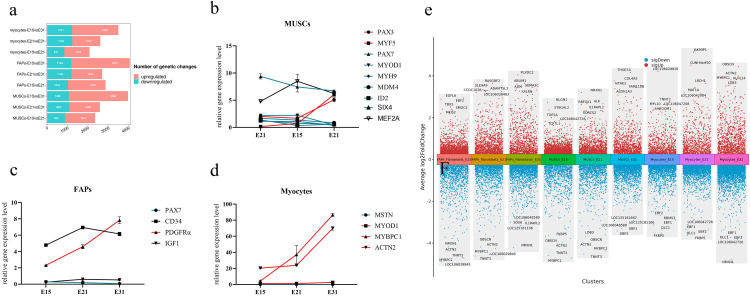
Expression levels of muscle cell genes in breast muscle during the embryonic development of goose. (a) The number of DEGs in myocytes at E15, E21, and E31 stages. (b) DEGs correlated with changes in the proportion of MUSC. (c) DEGs correlated with changes in the proportion of fibro-adipogenic progenitors (FAPs). (d) DEGs correlated with changes in the proportion of myocytes. (e) DEGs associated with brest muscle development.

To investigate the differences in transcriptional regulation during breast muscle development, we next analyzed changes in the transcriptional profile of MuSCs, FAPs, and myocytes. Analysis revealed that there were differences in the number of DEGs among the three cell types on E15, E21, and E31 (Fig 6a). E15 vs E31 had the largest DEGs number. Moreover, when analyzed across three time points, we found that the expression levels of *Pax7*, *SIX4*, *Mdm4*, *Id*, and *SIX4* decreased in MUSC, whereas the expression of *Myf5* was upregulated. Notably, the expression of *MEF2* reached its peak at E21 (Fig 6b). In FAPs, a reduction in *Pax7* expression was observed, accompanied by an upregulation of *PDGFRα*, *CD34*, and *IGF1* (Fig 6c). In myocytes, the expression levels of *MyoD*, *Mybpc*, and *Actn* were elevated, while *MSTN* expression was reduced (Fig 6d). Finally, we noticed that *ACTN2*, *MYBPC1*, *TNNT3*, and *LDB3* were significantly downregulated in MuSCs and FAPs (P < 0.01), and significantly upregulated (P < 0.01) in myocytes during the three developmental stages (Fig 6e).

## 4. Discussion

This study reports three key novel findings on embryonic breast muscle development in Ding’an goose. Muscle stem cells (MuSCs) can be subdivided into four functionally distinct subpopulations, MUSC-0 (proliferative, expressing TOP2A/MKI67), MUSC-1 (migratory, expressing MMP16/LAMA4), MUSC-2 (transcriptionally regulatory, expressing SOX13/TEAD4), and MUSC-3 (transcription factor-active, expressing ZBTB16/FOXO3)—which sequentially dominate early, middle, and late developmental stages. (Fibroblast adipogenic progenitor cells (FAPs) differentiate into a unique ‘muscle cell-like FAP’ subpopulation (expressing FOXO3/PLXDC2/ABLIM1) that becomes the dominant FAP subtype at E31, potentially participating in muscle fiber maturation. Myocytes undergo a subtype transition from Type IIa fiber dominance (E15-E21) to Type IIb fiber dominance (E31), which may underlie the breed’s high lean meat rate. These findings collectively delineate the cellular and molecular dynamics specific to Ding’an goose embryonic breast muscle development.

In the present study, we systematically explored the cellular heterogeneity and transcriptional characteristics of skeletal muscle at the single cell level from different aspects in Ding’an goose at E15, E21, and E31 for the first time. Nine cell types were identified in the breast muscle of goose, including myocytes, MuSCs, FAPs/fibroblasts, ECs, SCs, pericytes, IC, RBC, and MSCs. These findings are similar to the SnRNA-seq analysis of mouse breast muscle, as described previously [31]. Early developmental MuSCs accounted for the largest proportion of cell types, while the proportion of myocytes gradually increased during the course of development, accounting for the largest proportion (70%) at E31. Similar to previous study [32] in mice, MuSCs were activated and proliferated during breast muscle development in goose. Some cells retained their MUSC identity, while others differentiated into further dividing myoblasts, which further differentiated into myocytes. More importantly, we observed significant differences in the proportions of identified cell types, including MuSCs, FAPs/fibroblasts, and myocytes at different time points. These results suggest that cell types are relatively stable during this period, and that breast muscle function remains in a dynamic state of change.

Previous study has indicated that MuSCs exhibit high level of heterogeneity during the development of breast muscle [32]. In this study, we identified four MuSCs cell sub-types (MUSC-0, MUSC-1, MUSC-2, MUSC-3) and investigated their unique transcriptome profiles and corresponding functional characteristics. In the MUSC-0, we found that *TOP2A, MKI67*, and *CEMNP* were expressed at high levels. Research shows that these genes are known to be significantly associated with cell cycle processes and DNA replication [33–35]. Therefore, we infer that this cell sub-types represents a group of proliferating MuSCs. In the MUSC-1, we found that the *MMP16*, *LAMA4*, and *SRGAP1* were highly expressed, these genes are associated with cell migration [36–39]. Thus, it is possible that MUSC-1 may be related to cell migration and signal transduction. The MUSC-3 specifically expressed *ZBTB16*, *FOXO3* and *COL4A5*, these genes may affect the development of embryonic breast muscle in goose by regulating the synthesis of transcription enzymes [40,41]. In summary, different cell sub-types of MuSCs develop distinct function in breast muscle development.

Pseudo-time analysis is a research method that allows for the recognition and prediction of cell development trajectories [42]. In the present study, we used pseudo-time analysis to investigate the developmental trajectory of embryonic MuSCs in goose. We found that DEGs in different states exhibit dynamic expression patterns and were during the pseudotemporal differentiation process, suggesting their potential involvement in this process. The MUSC-0 plays a role in the early stages of embryonic development, while the MUSC-1, MUSC-2, and MUSC-3 play roles in the middle and late stages of development. The genes were highly expressed in States 1 and 2, and enriched in the cytoplasm and nucleus, while those in State 3 were associated with the overall composition of the cytoplasm and membrane. These results indicated that the metabolism of the MuSCs was enhanced, thereby supporting the growth and development of embryonic breast muscle in goose.

It is well-established that Fibroblast/adipocyte progenitor cells (FAPs) play an important role in muscle regeneration, the maintenance of homeostasis, injury response, and their relationship with adipose tissue [43]. Our analysis confirmed that the main cell sub-types of FAPs were FAPs-1, FAPs-2, FAPs-3, FAPs-4, FAPs-5, and muscle cell-like FAPs. Muscle cell-like FAPs specifically express *FOXO3*, *PLXDC2*, and *ABLIM1*, which are known to be associated with cell migration and cytoskeleton dynamics [44,45]. This may explain the proportion of muscle cell-like FAPs in the majority of cells during late embryonic development. Muscle fiber subtype is a core determinant of poultry meat quality. Type I and Type IIa fibers (slow-twitch fibers) rely on aerobic metabolism, which is associated with bright meat color, small fiber diameter, and high tenderness. Type IIb fibers (fast-twitch fibers) mainly use glycolytic metabolism, contributing to high lean meat content [46,47]. Ding’an goose is a local breed in Hainan Province known for its high lean meat rate (~75% in breast muscle) and good tenderness [20] Our results show that Type IIb fibers become the dominant subtype at E31 (90%), which is likely a key cellular basis for its high lean meat trait. Additionally, the maintenance of low but stable Type I fibers (<5%) and the gradual transition from Type IIa to Type IIb fibers may explain the good tenderness of Ding’an goose meat. Previous studies on goose have confirmed that moderate Type IIb fiber proportions avoid tough meat texture, which is consistent with consumer preferences for Ding’an goose [48]. This link between embryonic muscle fiber development and adult meat quality provides a targeted direction for optimizing Ding’an goose genetic breeding.

The development of breast muscle is the result of the synergistic effect of multiple cells [49]. Research has found that *Pax3* is associated with the transformation of muscle cells into limb muscles, and the inactivation of *Pax3* leads to the loss of the limb muscle system [50]. *Myf5* is the first activated muscle regulatory factor, and the inactivation of the *Myf5* gene leads to delayed formation of myotubes [51]. In this study, we found that the expression levels of *Myf5* and *Pax3* increased in MuSCs. Therefore, we speculate that *Myf5* and *Pax3* promote the transition of MuSCs to Myocytes during development. *Pax7* is involved in muscle regeneration and repair [52], *Mdm4* expression helps maintain the proliferation ability of MuSCs to support muscle regeneration [53], and *SIX4* inhibits the activation of slow muscle genes [54]. We found that the expression of *Pax7*, *Mdm4*, and *SIX4* were decreased during development, which inhibiting the formation of MuSCs. This is consistent with the previous findings that the proportion of MuSCs decreases during embryonic development. *Pax7* and *PDGFRα* are involved in regulating the proliferation and differentiation of FAPs [55]. Our findings revealed a downregulation of *Pax7* expression, accompanied by an upregulation of *PDGFRα* expression. *PDGFRα* may decrease the proportion of FAPs by inhibiting their proliferation. Research has found that *MyoD1* can induce multiple cell types to differentiate into Myocytes [56], *Mybpc* is involved in regulating muscle contraction efficiency [57], and *Actn* is involved in maintaining the skeletal structure and stability of Myocytes [58]. We found an increase in the expression of *MyoD*, *Mybpc*, and *Actn* in Myocytes. These genes promoted the transition of other cells to Myocytes, maintained the stability of Myocytes structure and function, and gradually made Myocytes occupy the largest proportion (70%) during development. It is noteworthy that biallelic pathogenic variants in *TNNT3* are associated with congenital myopathies [59], different splice isoforms of the *LDB3* have significant effects on chicken muscle atrophy and sarcomere formation [60], *MYBPC1* knockout mice exhibit impaired skeletal muscle formation and structure after birth [61], and *ACTN2* plays a pivotal role in muscle tissue and myopathies [62]. We found that *ACTN2*, *MYBPC1*, *TNNT3*, and *LDB3* were significantly downregulated in MUSC and FAP, while they were significantly upregulated in myocytes. These analyses revealed that DEGs that involved in regulating the growth and development of skeletal muscles in goose can serve as candidate genes related to the regulation of muscle growth and development in Ding’an goose.

This study has several limitations that should be noted. First, the snRNA-seq technology used in this study only captures nuclear transcriptomes, which may miss mRNAs highly enriched in the cytoplasm. This may lead to incomplete characterization of gene expression profiles in breast muscle cells. Future studies could combine single-cell RNA-seq (scRNA-seq, which captures whole-cell transcriptomes) with snRNA-seq to obtain more comprehensive gene expression information. Second, our analysis focused on three discrete time points (E15, E21, E31); more frequent sampling intervals would help refine the differentiation trajectory of MuSCs and FAPs, potentially identifying transient cell subtypes that play key roles in intermediate developmental stages. Third, we only verified the expression of key DEGs via qPCR and immunofluorescence, but did not validate their functional roles in breast muscle development. Such functional validation would further confirm the causal relationship between DEGs and cell fate decisions. Despite these limitations, the single-cell nucleus transcriptomic atlas of Ding’an goose embryonic breast muscle constructed in this study still provides a foundational resource for understanding the molecular mechanisms of goose breast muscle development.

## Conclusions

In summary, this study is the first to construct a single-cell nucleus transcriptomic atlas of breast muscle development in Ding’an goose embryos, revealing the heterogeneity of myoblasts, myocytes, and FAPs. This provides a comprehensive resource for understanding the characteristics, functions, and intercellular interactions of breast muscle cells in Ding’an goose. Additionally, we investigated differences in cell proportions and gene expression levels at the cellular level during key development stages (E15, E21, and E31) to reveal dynamic changes in cell composition and functionality during embryonic development, thus helping us to further understand the molecular mechanisms underlying breast muscle development in Ding’an goose.

## Supporting information

S1 Table snRNA Sequencing Metadata with Pseudotime Analysis for Goose Skeletal Muscle Cells. (XLSX)

## Abbreviations

DEGs: the differentially expressed genes
FAPs: fibroblast adipogenic progenitor cells/fibroblasts
SCs: Schwann cells
Peri: pericytes
Myocyte: muscle cells
ICs: immune cells
RBCs: red blood cells
ECs: endothelial cells
MuSCs: muscle stem cells
E15: goose the 15th day
E21: goose the 21th day
E31: goose the 31th day
